# Blotting Paper-Derived Activated Porous Carbon/Reduced Graphene Oxide Composite Electrodes for Supercapacitor Applications

**DOI:** 10.3390/molecules24244625

**Published:** 2019-12-17

**Authors:** Qinting Jiang, Dandan Liu, Bo Liu, Tong Zhou, Jin Zhou

**Affiliations:** 1 Lab of Functional Molecules and Materials, School of Physics and Optoelectronic Engineering, Shandong University of Technology, Zibo 255049, China; Qintingjiang@163.com (Q.J.); liub@sdut.edu.cn (B.L.); 2 School of Chemistry and Chemical Engineering, Shandong University of Technology, Zibo 255049, China

**Keywords:** biomass-derived activated carbon, reduced graphene oxide, supercapacitor

## Abstract

A facile strategy, engineered for low-cost mass production, to synthesize biomass-derived activated carbon/reduced graphene oxide composite electrodes (GBPCs) by one-pot carbonization of blotting papers containing graphene oxide (GO) and zinc chloride (ZnCl_2_) was proposed. Benefitting from the water absorption characteristic of blotting papers in which the voids between the celluloses can easily absorb the GO/ZnCl_2_ solution, the chemical activation and reduction of GO can synchronously achieve via one-step carbonization process. As a result, the GBPCs deliver a large specific surface area to accumulate charge. Simultaneously, it provides high conductivity for electron transfer. The symmetric supercapacitor assembled with the optimal GBPCs in 6 M KOH electrolyte exhibits an excellent specific capacitance of 204 F g^−1^ (0.2 A g^−1^), outstanding rate capability of 100 F g^−1^ (20 A g^−1^). Meanwhile, it still keeps 90% of the initial specific capacitance over 10,000 cycles. The readily available raw material, effective chemical activation, simple rGO additive, and resulting electrochemical properties hold out the promise of hope to achieve low-cost, green, and large-scale production of practical activated carbon composite materials for high-efficiency energy storage applications.

## 1. Introduction

As the most promising energy storage devices, supercapacitors have become an ever-increasing demand for applications in advanced hybrid powers, energy backups, and pulsed power devices owing to their merits of energy density greater than traditional capacitors and power output higher than conventional batteries [1,2,3,4,5]. On the basis of the electrostatic storage by physical adsorption of electrolyte ions or faradaic redox reactions with the charge-transfer, supercapacitors can be divided into electrical double-layer capacitors (EDLCs) and pseudocapacitors [6,7,8,9,10], both of which have their own advantages: the former possesses a higher power density with an excellent cycling stability [11,12,13], while the latter exhibits a larger energy density but more rapid loss [14,15,16]. To promote the development of supercapacitors, the urgent issue is to design high-performance and low-cost electrode materials [7,17,18,19]. Considering the severe capacitance fading and poor cycling stability of pseudocapacitors, EDLCs stand much closer to the practical applications due to their performance stability [18,20,21,22]. Advanced carbon materials, as one of the ideal electrode material for EDLCs, ensure high power output and energy density because of its high electrical conductivity and large surface area [7,23,24,25].

Rich raw material and easy synthesis make carbon materials successful in large-scale production. Compared with non-renewable petrochemical resources, sustainable biomass wastes (usually composed of cellulose, hemicellulose, and lignin) are naturally abundant in every corner of our lives, which has an advantage in producing low-cost electrode materials [26,27,28,29,30]. Until now, biomass-derived carbon materials based on carbonization of corn straw [31], jujube [32], filter paper [33], sugarcane bagasse [34], cotton [35], and rice husk [36] have been fully exploited as the electrode materials for supercapacitor applications. However, the pore-forming occurred in the pyrolysis of biomass waste is insufficient that the specific surface area (SSA) is still too small. To obtain biomass-derived carbon materials with a high SSA, it is essential to carry out a suitable pore-forming treatment by adding the activation agents [23,37,38]. With respect to the physical activations using steam or CO_2_, the chemical activations involving KOH, H_3_PO_4_, and ZnCl_2_ can give the porous pyrolytic carbon an ultrahigh SSA [8,39,40,41,42]. However, the activation process significantly destroys the carbon frameworks, which is adverse to the enhancement of power density due to the decline of the electrical conductivity of electrodes [11]. For addressing such a bottleneck, embedding conductive additives into the porous carbon is used for the electrode preparation. Contrary to other conductive additives (graphite nanoparticles, carbon nanotube, and carbon onion), graphene has been successfully applied in electrochemical energy storage devices owing to its extraordinary electrical conductivity and excellent electrochemical stability [43,44,45]. Among the various methods to synthesize graphene, the oxidation–exfoliation–reduction of graphite powers appears to be the simplest one for mass applications [46]. Thereby, in order to exploit all the above merits more adequately, a facile strategy for the preparation of composite materials combined biomass carbon and graphene needs to be developed urgently [47,48].

Herein, we present a one-step method for incorporating graphene oxide (GO) and zinc chloride (ZnCl_2_) into blotting papers via its self-absorption characteristic. Then, these mats were heat-treated to emerge the composite electrode materials (graphene oxide composite electrodes (GBPCs)) with activated porous carbon and thermally reduced graphene oxide (rGO). The morphology, microstructure, and porosity characteristics of GBPCs were investigated by scanning electron microscopy, high-resolution transmission electron microscopy, N_2_ adsorption/desorption, Raman spectroscopy, and X-ray diffraction, all of which suggest that the GBPCs is an advanced electrode material for supercapacitor. The electrochemical measurements reveal that the optimal GBPCs exhibit a remarkable capacitance of 204 F g^−1^ and a good rate capability (remains 100 F g^−1^ at 20 A g^−1^). Meanwhile, it still keeping 90% of the initial specific capacitance over 10,000 cycles. These highlight the promise of the carbons for high power applications in all aspects.

## 2. Results and Discussion

### 2.1. Characterizations of GBPCs

**Figure 1:**
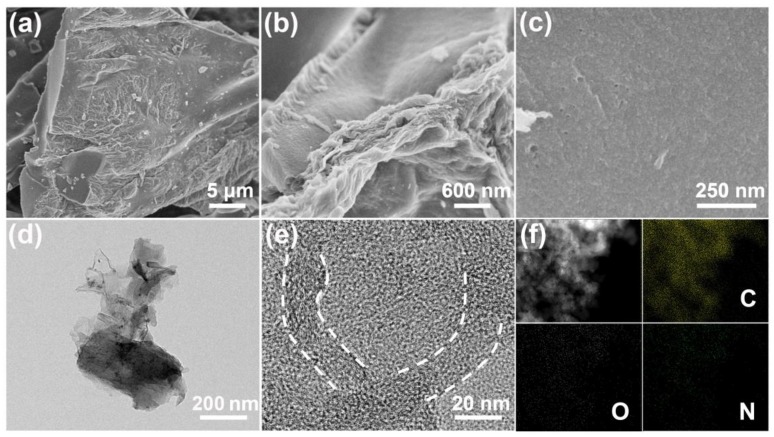
(**a**–**c**) SEM images of the graphene oxide composite electrode (GBPC)-1, (**d**,**e**) TEM images of GBPC-1, and (**f**) STEM-EDS mapping of C, N, and O elements in GBPC-1.

The morphologies and microstructures of GBPC-1 were studied by SEM and TEM, respectively. As shown in Figure 1a, micro-sized porous carbon particles were derived from the blotting papers after the pyrolysis process and chemical activation, which destroy paper integrity. A smooth fracture surface of the carbon particle could be clearly seen. However, the partial surface of the carbon particle shows some wrinkles. The wrinkles on the surface of carbon particles are actually due to the coating of the wrinkled rGO, which is verified by the stacking of rGO sheets as displayed in Figure 1b. As shown in the high-magnification SEM image of the smooth fracture surface (Figure 1c), plenty of pores can be clearly visible. It is proved that the pyrolytic carbon has been corroded after the ZnCl_2_ activation. As revealed by Figure 1d, the TEM image of GBPC-1 clearly shows that the corresponding carbon particle has been largely wrapped by the rGO sheets. As shown in Figure 1e, the curved stacking layers of the rGO sheets are further revealed by the high-resolution TEM image. The high conductivity of rGO and the porous network of pyrolytic carbon can provide both valid electron transport paths and ion transfer pathways that a much better rate performance and a much higher capacitance can be expected in the supercapacitor application. In addition, the characterization of GBPC-1 was also performed by STEM-EDS mapping. The results are shown in Figure 1f. It is clearly shown that the GBPC-1 contains C, N, and O elements without any other impurity element.

**Figure 2:**
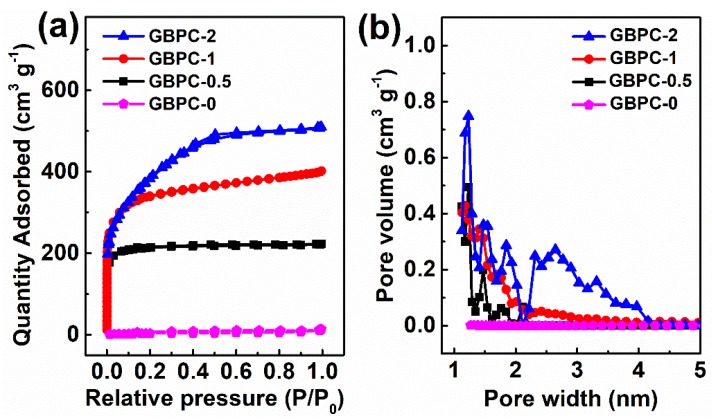
(**a**) N_2_ sorption isotherms and (**b**) pore size distribution of the GBP.

The N_2_ adsorption and desorption measurements were also performed to determine the specific surface area and pore structure of GBPCs (Figure 2). Compared with other GBPCs, the adsorbed quantity of GBPC-0 was quite small. The BET specific surface area of GBPC-0 was only 13.4 m^2^ g^−1^, suggesting that very few pores were introduced during the pyrolysis process. Compared with the GBPC-0, the GBPC-0.5 possessed a high adsorbed quantity at the low relatively pressure (P/P_o_ < 0.02). It could be seen that the GBPC-0.5 exhibited a type I isotherm, indicating that there was a large number of micropores resulting in the adsorbate filling at the low relative pressure [39]. For the GBPC-1, its N_2_ sorption isotherm was upturned at the relative pressures higher than 0.02, indicating that there were plenty of mesopores in the carbon framework. As the ZnCl_2_ dosage increased, the adsorbed quantity further increased. The porosity parameters of GBPCs are present in Table 1. With the rise of ZnCl_2_ dosage, the BET specific surface area of GBPCs increased from 715.7 to 1735.6 m^2^ g^−1^, and pore volume increased from 0.34 to 1.12 cm^3^ g^−1^, indicating the high efficiency of ZnCl_2_ on developing porosity. The process of pore-forming can be roughly described that the ZnCl_2_-activation firstly developed micropores and then widened the micropore to mesopores at high ZnCl_2_ doses. Meanwhile, with the development of the porosity of GBPCs, the specific surface area of micropores is to be maximized for the GBPC-1.

**Figure 3:**
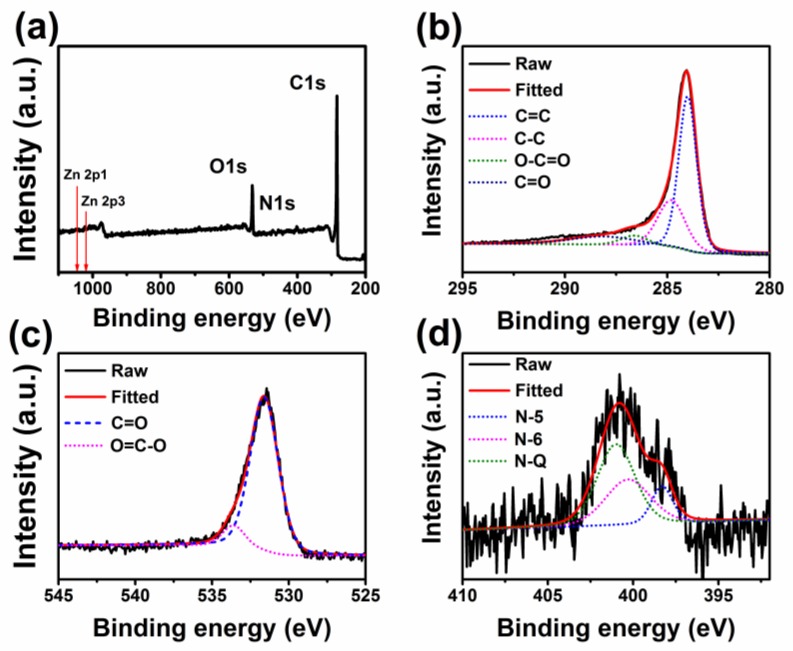
(**a**) XPS survey, high-resolution XPS spectra of (**b**) C1s, (**c**) O1s, and (**d**) N1s of GBPC-1.

To determine the composition and chemical state of the elements in GBPC-1, X-ray photoelectron spectroscopy (XPS) testing was used. In Figure 3a, there are three peaks of C1s (284 eV), O1s (532 eV), and N1s (399 eV) of GBPC-1 coexisting [11]. Simultaneously, we did not find Zn element in the XPS survey of GBPC-1, meaning that the Zn element has been removed completely by HCl solution. High-resolution of the C1s spectrum in Figure 3b can be deconvolved into four separate component peaks, which correspond to C=C (284.8 eV), C-C (285.6 eV), O-C=O (287.4 eV), and C=O (288.9 eV), respectively. From the deconvolution results, we can see the existence of C=C, which not only proves the existence of rGO, but also confirms that rGO is wrapped on the surface of amorphous carbon. This result is consistent with the result of TEM. The atomic percentages of O and N in GBPC-1 are 11.36% and 2.79%, respectively. The O1s spectrum (Figure 3c) can be deconvoluted into four individual component peaks corresponding to C=O (531.6 eV) and O=C-O (533.6 eV). These highly O-doped heteroatoms can improve the wettability between the electrode and the electrolyte, thereby enhancing the electrochemical performance of the electrode. Besides, the test results show that it contains N elements. The N elements are mainly from cellulose, the main component of blotting paper, that contains certain nitrogen, which was reported in previous works [46,49,50]. As shown in Figure 3d, the atomic binding state of N in GBPC-1 was equipped with four components corresponding to pyridine N (N-6), pyrrole N (N-5), and quaternary N (N-Q), respectively. Among these, N-5 and N-6 substances are considered to be electrochemically active sites of the reversible redox reaction.

**Figure 4:**
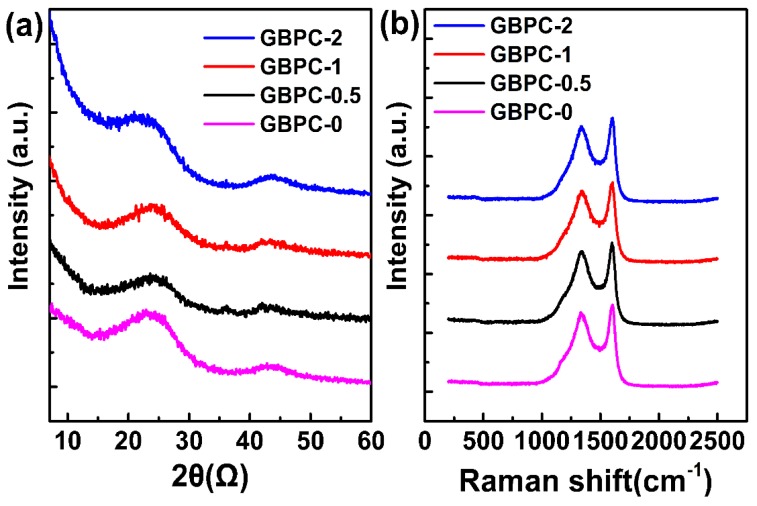
(**a**) XRD patterns and (**b**) Raman spectra of GBPCs.

Figure 4 shows the XRD patterns and Raman spectra of GBPCs. For all of the GBPCs, two broad diffraction peaks located at 2θ = 24.2° and 43.5°, which correspond to the (002) and (101) planes of carbon material, are discernibly observed, revealing the characteristics of amorphous carbon [44]. The intensity of the diffraction peak decreases seriously with the ZnCl_2_ increasing, further confirming the activation effect on the GBPCs. The diffraction peak of GO (2θ = 11°) also was not observed, indicating that the GO sheets have been effectively reduced. Raman spectroscopy was also used to investigate the disorder of GBPCs. There were two characteristic peaks, one of which was the D-band located at 1340 cm^−1^, and the other was the G-band centered at 1589 cm^−1^ [11]. The degree of amorphization of GBPCs can be evaluated by the intensity ratio of D-band and G-band. The intensity ratio of I_D_/I_G_ was 0.902, 0.906, 0.906, and 0.912 for the GBPCs with the increasing ZnCl_2_ dose.

### 2.2. Electrochemical Performance in 6 M KOH Electrolyte

**Figure 5:**
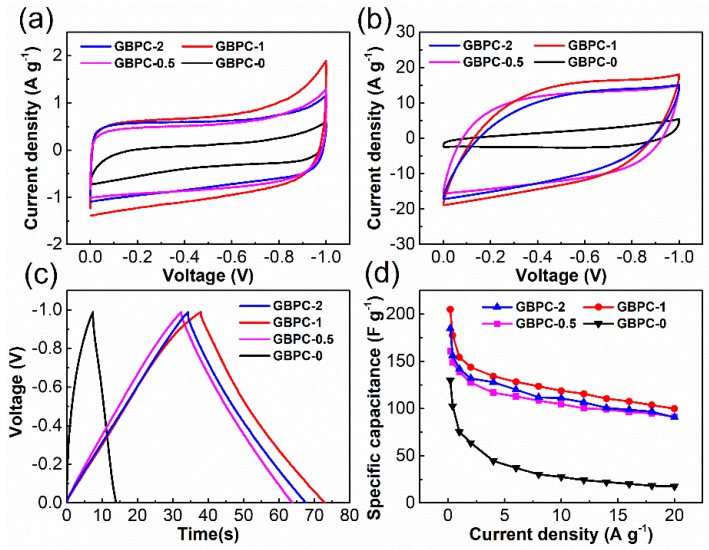
Electrochemical performance of GBPCs in 6 M KOH electrolyte: cyclic voltammetry (CV) curves (**a**) at 5 mV s^−1^, (**b**) at 300 mV s^−1^, (**c**) galvanostatic charge/discharge (GCD) curves at 2 A g^−1^, and (**d**) specific capacitance at current densities from 0.2 to 20 A g^−1^.

Electrochemical behaviors of all the GBPCs were measured with a two-electrode system in an electrolyte of 6 M KOH. For the ideal electrical double-layer capacitors, the shape of CV curves should be a rectangle as the standard at a constant sweep rate. At the same scan rate, the enclosed area of the CV curve could stand for the ability of a capacitor to store charges. Figure 5a shows the CV curves of GBPCs electrodes at the scan rate of 5 mV s^−1^ in the voltage range from –1 to 0 V. All the CV curves of GBPCs exhibit an approximately rectangular shape, indicating that the capacitive performances are primarily determined by electrochemical double-layer capacitance. In contrast, the GBPC-1 has the highest specific capacitance, the GBPC-0.5 and GBPC-2 are the second, and the GBPC-0 is the least. When the scan rate was as high as 300 mV s^−1^, the CV curve of GBPC-0 was far from its primeval rectangular shape. Especially at the charging part, the current density of GBPC-0 was nearly proportional to the voltage, meaning that the GBPC-0 had lost its capacitance characteristic and shows a resistive behavior. At the same condition, the CV curve of GBPC-1 kept throwing a rectangular shape well, indicating the superior capacitance retention of the GBPC-1.

Galvanostatic charge/discharge (GCD) measurements are one of the common methods to study the capacitive performance of electrode materials. Figure 5c shows the GCD curves of the GBPC-0, GBPC-0.5, GBPC-1, and GBPC-2 at a current density of 2 A g^−1^. It can be seen that the GCD curve of GBPC-1 exhibited an isosceles triangle-like shape, confirming that the energy storage mechanism of the electrode material was mainly the double-layer energy storage. It is also known that the charge and discharge time of GBPC-1 was the largest one compared with others. The capacitor performance of GBPC-1 was proved to be the best. This result was consistent with the results of the CVs. We also provided the charge–discharge profile of GBPC-1 from 0.2 to 20 A g^−1^ in Figure S1.

The specific capacitances of GBPCs calculated from the discharging time of GCD curves under different current densities are shown in Figure 5d. The specific capacitance of GBPC-1 was 205 F g^−1^ at the current density of 0.2 A g^−1^, which was 58%, 27%, and 11% higher than the values of GBPCs-0, GBPCs-0.5, and GBPCs-2. The specific capacitance of all the GBPCs decreased with the increase of the current density. This is because the shortened charging time makes the electrolyte ions not completely diffuse into the micropores that drops the specific capacitance. When the current density was increased by 100 times to 20 Ag^−1^, the specific capacitance of GBPC-1 could still reach 100 F g^−1^, showing a considerable rate capability with a capacitance retention of about 50%. The superior capability of GBPC-1 in capacitive energy storage could be attributed to the following synergistic effect by two factors. One factor is the increased micropore surface area through ZnCl_2_ activation that promotes the electrostatic storage by physical adsorption of electrolyte ions. The other is the highly conductive rGO, which facilitates electron transport, thus leading to remarkably improved rate capability. In addition, to investigate the role of rGO and ZnCl_2_, we prepared two additional samples without any GO content BCP-0 and BCP-1, and tested their electrochemical performance. As shown in Figure S2, the addition of rGO and ZnCl_2_ could both improve the specific capacitance of the blotting papers material and its capacitance retention rate under high current density. The electrochemical performance of GBCP-1 was the most outstanding, which also indicates that the excellent performance of GBCP-1 came from the synergy of rGO and ZnCl_2_. The Nyquist plots of BPC-0, GBPC-0, BPC-1, and GBPC-1 are clearly shown in Figure S3. We can see that the semi-circular arcs of GBPC-0 and BPC-1 in the intermediate frequency region were smaller than BPC-0, and the semi-circular arc of GBPC-1 was the smallest, indicating that electron transmission rate of GBPC-1 was the fastest. This proved from another aspect that the adding of rGO and ZnCl_2_ both contributed to the improvement of the electron transfer rate, and the excellent performance of c is the joint effect of rGO and ZnCl_2_.

**Figure 6:**
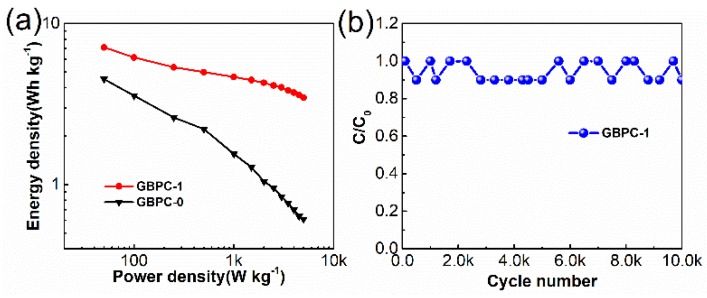
(**a**) Ragone plots of GBPC-0 and GBPC-1 and (**b**) cycling stability of GBPC-1 at a current density of 5 A g^−1^.

Figure 6a shows the Ragone curves of the GBPC-0 and GBPC-1, respectively. Notably, the GBPC-1 delivers an energy density of 7.1 Wh kg^−1^ at the power density of 50 W kg^−1^, which is much higher than the case of GBPC-0 (4.5 Wh kg^−1^ at 50 W kg^−1^). Meanwhile, the energy density of GBPC-1 remained 3.5 Wh kg^−1^ at a power density of 5000 W kg^−1^, while the energy density of the GBPC-0 was only 0.61 Wh kg^−1^. Obviously, the energy density of GBPC-1 had superior energy storage and power output. As shown in Figure 6b, the cycle stability of GBPC-1 was evaluated by the continuous GCD cycling at 5 A g^−1^. With the increase of the cycle number, the capacitance of GBPC-1 slightly decreased, which might be caused by the decomposition of certain unstable functional groups in the process of charge/discharge cycle, resulting in the loss of some pseudo-capacitor. After 10,000 cycles, the capacitance of GBPC-1 still maintained 90% of its initial capacitance, manifesting the excellent cyclic stability. Above all, we could see that GBPC-1 shows excellent electrochemical performance, and the comparison of the capacitive performance of other biomass-based thermal annealed electrodes is presented in Table S1 [51,52,53,54,55,56,57,58,59,60].

**Figure 7:**
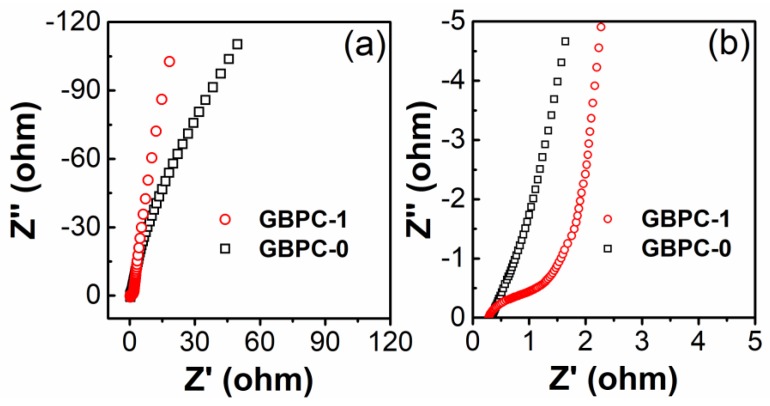
(**a**) The complete and **(b)** high-frequency region of electrochemical impedance spectra of GBPCs-1 and GBPCs-0.

Furthermore, the electrochemical impedance spectroscopy (EIS) of GBPCs was measured to analyze the electrochemical behavior of GBPCs, which the frequency ranged from 0.001 to 100,000 Hz. The Nyquist plots of GBPC-0 and GBPC-1 are clearly shown in Figure 7a. The GBPC-1 exhibited a steeper linear slope at the low-frequency region, implying its almost ideal capacitive behavior [7]. Compared with the GBPC-1, the linear slope of GBPC-0 gravely inclined to the real axis because of its kinetic limitation, explaining its bad rate capability. As shown in the magnified Nyquist plot at the high-frequency region (Figure 7b), an obvious semicircle was observed in the case of GBPC-1, indicating that the presence of porous structure facilitated a rapid charge transfer between the electrolyte and electrode due to the increased electrode surface area.

## 3. Materials and Methods

### 3.1. Chemicals and Materials

The blotting papers were collected from our daily life. Graphite flakes were obtained from Sigma-Aldrich Co., Ltd.(Shanghai, China). Analytical Reagents of sodium nitrate (NaNO_3_, ≥99.0%), zinc chloride (ZnCl_2_, ≥98.0%), potassium permanganate, (KMnO_4_, ≥99.5%), sulfuric acid (H_2_SO_4_, 95.0–98.0%), hydrogen peroxide (H_2_O_2_, ≥30.0%), and hydrochloric acid (HCl, 36.0–38.0%) were all purchased from Sinopharm Chemical Reagent Co., Ltd. (Shanghai, China). Deionized (DI) water was prepared by a water purification system with a resistance of 18.2 MΩ cm^−1^.

### 3.2. Preparation of Graphene Oxide

A modified Hummers method was taken to make GO, by which the commercial graphite flakes were chemically oxidized and then ultrasonically exfoliated into monolayer flakes of GO. Briefly, graphite flakes (2 g) was added into the mixture of concentrated H_2_SO_4_ (96 mL) and NaNO_3_ (2 g) in an ice bath under vigorous stirring. Then, KMnO_4_ (12 g) was added gradually into the above mixture. After stirring for 1.5 h, the temperature of the mixture was elevated to 35 °C, followed by a subsequent stir for 2.5 h. Then, repetitive actions were carried out at 55 °C for 1.5 h. Subsequently, DI water (200 mL) and H_2_O_2_ (10 mL) were dropwise added, respectively. Finally, the yellow GO dispersion (5 mg mL^−1^) was obtained by repeatedly washing the mixture with DI water.

### 3.3. Synthesis of GBPCs

Firstly, ZnCl_2_ power was mixed evenly into the as-prepared GO solution. Next, we soaked blotting papers into the mixed solution for absorbing the GO and ZnCl_2_ via the self-absorption of blotting papers. It is worth mentioning that the mass ratio of GO and blotting papers was 1:9, and then the mixed solution was completely absorbed by blotting papers. Then, the samples were completely dried in an electro-thermostatic blast oven at 80 °C. Subsequently, the resultant materials were calcined at 700 °C for 2 h under the N_2_ protection. Finally, the resulting samples were thoroughly washed with 1 M HCl solution to eliminate the non-carbon sources and rinsed with DI water to neutrality. For convenience, the activated porous carbon samples were named as GBPC-x, where x (x = 0, 0.5, 1, or 2) stands for the weight ratio of ZnCl_2_ over blotting papers. For comparison, the samples of blotting papers and ZnCl_2_ without GO were also prepared, named BCP-x.

### 3.4. Material Characterization

The morphology of GBPCs was characterized by using a field emission scanning electron microscope (FESEM, Sirion 200, FEI, Hillsboro, OR, USA) and high-resolution transmission electron microscopy of GBPCs was acquired using the transmission electron microscope (HRTEM, H800, Hillsboro, OR, USA). The chemical compositions of GBPCs were examined using X-ray photoelectron spectroscopy (XPS, Escalab250). The crystal properties of GBPCs were executed by X-ray diffraction patterns (XRD, Brucker, AXS, Karlsruhe, Germany, D8 Advance diffraction with Cu Ka radiation) and Raman scattering spectra (Horiba, LabRam HR spectrometer, Kyoto, Japan), respectively. Nitrogen sorption measurements were performed at liquid nitrogen temperature using an ASAP 2020 system (Micrometitics, Quantachrome, FL, USA).

### 3.5. Electrochemical Measurements

The as-prepared GBPCs were thoroughly mixed with polytetrafluoroethylene (PTFE) binder at a weight ratio of 95:5 to form a slurry. Then, the slurry was rolled into an electrode film and dried at 120 °C for 12 h. Last, the electrode film was sandwiched between nickel foams under 10 MPa pressure to form a slice. The mass loading and geometric area of the electrode material on the working electrode was about 2 mg and 0.25 cm^2^ (0.5 cm × 0.5 cm), respectively, responding to about 8 mg cm^−2^. The electrochemical measurements were carried out on a CHI760E electrochemical testing station (Chenhua Instruments Co. Ltd., Shanghai, China). The electrochemical tests in 6 M KOH electrolyte using a two-electrode system, including cyclic voltammetry (CV) and galvanostatic charge/discharge (GCD) measurements. The two-electrode system is packaged in a 2032 coin cell consisted of two work electrodes and a sandwiched cellulose separator. The long-term GCD experiment was carried out at an automatic galvanostatic charge/discharge device (Land CT 2001 A, Wuhan, China) under a current density of 5 A g^−1^. The specific capacitance of as-prepared electrodes was determined from the GCD tests, by the following formula,

(1) $$C_{m}=\frac{2I\times t}{\Delta V\times m},$$

where Cm (F g^−1^) is the gravimetric specific capacitance of GBPC, I (A) is the discharge current and t (s) and is the discharge time, ∆V (V) is the potential window, and m (g) is the mass loading of a single electrode.

The energy density (E, Wh kg^−1^) and power density (P, W kg^−1^) could be calculated from the equations below:

(2) $$E=\frac{C_{m}\times V^{2}}{8}.$$

(3) $$P=\frac{E}{t}.$$

## 4. Conclusions

In summary, we proposed a facile strategy to prepare porous carbon materials from the blotting papers containing the GO and ZnCl_2_ solution. During the carbonization process, chemical activation, and reduction of GO can be synchronously achieved. For the supercapacitor application, the optimal GBPCs exhibited a high specific capacitance, good rate capability, and excellent cycling stability in 6 M KOH electrolyte, owing to its large specific surface area for accumulation of charges and well electrical conductivity for electron transfer. The readily available raw material, effective chemical activation, simple rGO additive, and resulting electrochemical properties take a promising prospect in realizing high-performance electrode with conventional porous carbon for high-efficiency energy storage applications.

## Figures and Tables

**Table 1:** Porosity parameters of GBPCs.

Sample	S_BET_ /m^2^ g^−1^	S_micro_ /m^2^ g^−1^	V_T_ /cm^3^ g^−1^	V_micro_ /cm^3^ g^−1^	V_macro_ /cm^3^ g^−1^	D /nm
GBPC-0	13.4	3.73	0.02	0.001	0.0019	5.97
GBPC-0.5	715.7	416.87	0.34	0.32	0.02	1.93
GBPC-1	1066.9	613.59	0.62	0.33	0.29	2.33
GBPC-2	1388.1	541.96	0.79	0.36	0.43	2.27

## Supplementary Materials

The following are available online, Figure S1: GCD curves of GBCP-1 from 0.2 A g^−1^ to 20 A g^−1^, Figure S2: Specific capacitance at current densities from 0.2 A g^−1^ to 20 A g^−1^, Figure S3: Electrochemical impedance spectra of GBPCs-1, BPC-1, GBPC-0, and GBPCs-0, Table S1: Comparison of the capacitive performance of other biomass-based thermal annealed electrodes.
