# Supplementary material for: Blotting Paper-Derived Activated Porous Carbon/Reduced Graphene Oxide Composite Electrodes for Supercapacitor Applications
Source: Molecules. 2019 Dec 17;24(24):4625. doi: 10.3390/molecules24244625 (PMC6943556; doi:10.3390/molecules24244625)
Supplement: Supplementary file 1 [file molecules-24-04625-s001.pdf]

Supporting Information

Blotting Paper-derived Activated Porous Carbon/Reduced Graphene Oxide Composite Electrodes for Supercapacitor Applications

Qinting Jiang [1,†], Dandan Liu [2,†], Bo Liu [1], Tong Zhou [1,*] and Jin Zhou [2,*]

[1] Lab of Functional Molecules and Materials, School of Physics and Optoelectronic Engineering, Shandong University of Technology, Zibo 255049, China; Qintingjiang@163.com (Q.J.); liub@sdut.edu.cn (D.L.); zhoutong@sdut.edu.cn (B.L.)

[2] School of Chemistry and Chemical Engineering, Shandong University of Technology, Zibo 255049, P. R. China; l834428148@163.com

* Correspondence: zhoutong@sdut.edu.cn (T.Z.); zhoujin@sdut.edu.cn (J.Z.); Tel.: +0533-278-7863 (T.Z.)

† These authors contributed equally to the work

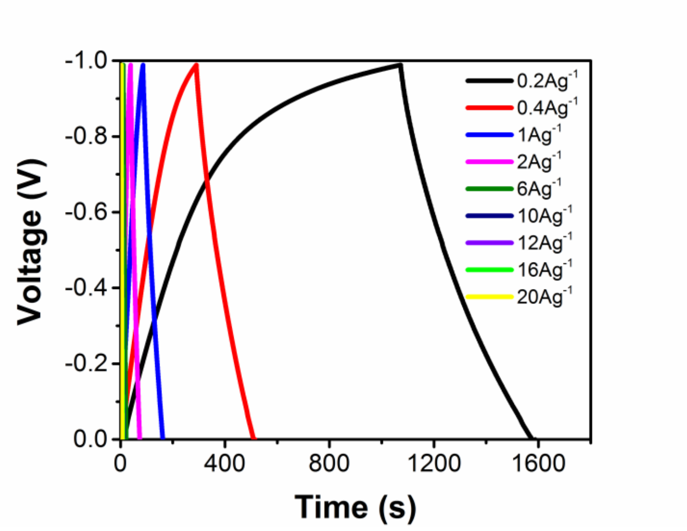

Figure S1. GCD curves of GBCP-1 from 0.2 A g^{-1} to 20 A g^{-1}.

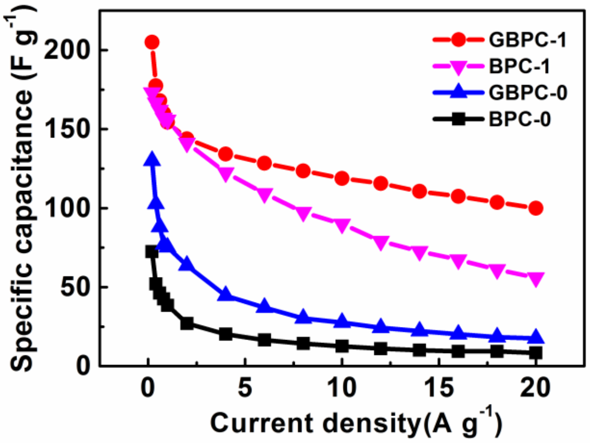

Figure S2. specific capacitance at current densities from 0.2 A g^{-1} to 20 A g^{-1}.

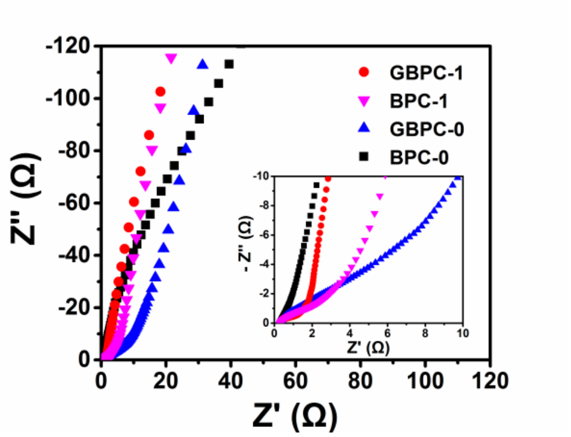

Figure S3. Electrochemical impedance spectra of GBPCs-1, BPC-1, GBPC-0, and GBPCs-0.

Table S1 Comparison of the capacitive performance of other biomass-based thermal annealed electrodes.

Material	Surface Area	Electrochemical Performance	Ref.
Cotton/rGO	392.6 $m^2 g^{-1}$	120F g^{-1} at 3 A g^{-1} in PVA/ KOH	[51]
Bacterial cellulose/rGO	137 $m^2 g^{-1}$	216 F g^{-1} at 0.1 A g^{-1} in 6.0 M KOH	[52]
Silk/GO	1927 $m^2 g^{-1}$	52.8 F g^{-1} at 0.18 A g^{-1} in 6.0 M KOH	[53]
Lignin/rGO	1744 $m^2 g^{-1}$	165 F g^{-1} at 20 A g^{-1} in 1.0 M H_2SO_4	[54]
Basewood	1438 $m^2 g^{-1}$	135 F g^{-1} at 0.2 A g^{-1} in 4.0 M KOH	[55]
Gelatin	50.8 $m^2 g^{-1}$	168 F g^{-1} at 5 A g^{-1} in 2.0 M Na_2SO_4	[56]
Peanut shell	2070 $m^2 g^{-1}$	186 F g^{-1} at 0.5 A g^{-1} in 1.0 M H_2SO_4	[57]
Chitin	1000 $m^2 g^{-1}$	138 F g^{-1} at 0.92 A g^{-1} in 1.0 M H_2SO_4	[58]
Sisal leaves	1137 $m^2 g^{-1}$	204 F g^{-1} at 1 A g^{-1} in 1.0 M LiOH	[59]
Viscose cloth	1205 $m^2 g^{-1}$	121 F g^{-1} at 0.02 A g^{-1} in 1.0 M H_2SO_4	[60]
Blotting paper/rGO	1066.9 $m^2 g^{-1}$	204 F g^{-1} at 0.2A g^{-1} in 6.0 M KOH	This work
